# Modeling Transmission Dynamics of Tuberculosis–HIV Co-Infection in South Africa

**DOI:** 10.3390/epidemiologia4040036

**Published:** 2023-10-10

**Authors:** Simeon Adeyemo, Adekunle Sangotola, Olga Korosteleva

**Affiliations:** 1 Department of Mathematics and Statistics, California State University, Long Beach, CA 90840, USA; olga.korosteleva@csulb.edu; 2 Department of Physical Sciences, Bells University of Technology, Ota 112212, Ogun, Nigeria; aosangotola@bellsuniversity.edu.ng

**Keywords:** tuberculosis, HIV, co-infection dynamics, mathematical modeling, South Africa

## Abstract

South Africa has the highest number of people living with the human immunodeficiency virus (HIV) in the world, accounting for nearly one in five people living with HIV globally. As of 2021, 8 million people in South Africa were infected with HIV, which is 13% of the country’s total population. Approximately 450,000 people in the country develop tuberculosis (TB) disease every year, and 270,000 of those are HIV positive. This suggests that being HIV positive significantly increases one’s susceptibility to TB, accelerating the spread of the epidemic. To better understand the disease burden at the population level, a Susceptible–Infected–Recovered–Dead (SIRD) TB–HIV co-infection epidemic model is presented. Parameter values are estimated using the method of moments. The disease-free equilibrium and basic reproduction number of the model are also obtained. Finally, numeric simulations are carried out for a 30-year period to give insights into the transmission dynamics of the co-infection.

## 1. Introduction

Tuberculosis (TB) is an infectious disease caused by the pathogen *Mycobacterium tuberculosis*. The bacteria are transmitted from the lungs of an active individual to the respiratory tracts of uninfected individuals by droplet nuclei that contain *M. tuberculosis* and are dispersed by the air–capillary route. The TB bacteria can cause infection without always leading to symptoms of illness. As a result, two conditions are associated with TB: latent TB infection (LTBI) and TB disease. People who have LTBI are asymptomatic and have the bacteria but are unable to pass it on to others. According to the World Health Organization, one-third of the world’s population is infected, either latently or actively, with TB [1]. TB is a serious public health issue in South Africa. About 450,000 people develop the disease every year, and 270,000 of those are also living with HIV. TB is South Africa’s leading cause of death. About 89,000 people die from it every year; that is ten people every hour [2]. This suggests that being HIV positive significantly increases one’s susceptibility to TB, accelerating the spread of the epidemic. Additionally, the huge difference in TB infections between persons with and without HIV infection remains a global challenge as a result of the high incidence rate among HIV-infected individuals [3]. TB and HIV can also coexist with diseases such as malaria and COVID-19 [4,5]. Effective treatments are available, and the country has made considerable progress in fighting the disease, but much more is needed to bring it under control. TB has slow intrinsic dynamics. The incubation period, latent period, and infectious period span long time intervals, in the order of years on average. The slow progression of TB at the individual level leads to slow temporal dynamics and long-term outcomes at the population level. Therefore, mathematical models are needed to estimate prolonged results, and future trends, and develop a better understanding of the epidemic [6,7,8].

For a long time, compartmental models have been useful in the mathematical modeling of infectious diseases. The Susceptible–Infectious–Recovered (SIR) model was developed by William Kermack and Anderson McKendrick [9]. SIR models, also known as Kermack–McKendrick models, depict the various states an individual can be in when exposed to an epidemic. SIR models include states that are not compatible with one another; therefore, individuals in the population can only be in one of those states at any given time. As the epidemic spreads, people may relocate from one compartment to another depending on their current illness level. Basic SIR models account for two types of transitions: the transition from susceptible (S) to infectious (I) and that from infectious (I) to recovered (R). The rate at which someone moves from S to I is called the rate of infection. This rate represents individuals moving from S to I after coming into contact with the disease. The rate at which an infected individual moves into the recovered population is referred to as the rate of recovery. 

There exist several models of the co-infection of TB/HIV depending on the research directive and objective. Awoke and Kassa [10] formulated a mathematical model for the transmission of TB–HIV/AIDS co-infection that incorporates prevalence-dependent behavior change in the population and treatment for the exposed and infectious. The two sub-models of the individual disease were analyzed. Azeez et al. [11] formulated the TB and HIV mathematical model to give more insight and to forecast the spread of the two infectious diseases in different populations. One major conclusion was that a community endemic with TB without undergoing treatment is at a greater risk of HIV co-infection. Ali et al. [12] investigated HIV/AIDS and TB within a population of varying size using a nonlinear model by incorporating media coverage as a form of awareness. Similar work was carried out by [13] but in the presence of protection as a form of control. Agusto and Adekunle [14] formulated an optimal control strategy for the mathematical modeling of HIV/AIDS co-infection. The impact of the several control combinations on the disease spread was also examined. 

Additional work on TB/HIV co-infection can be seen in [15,16,17,18,19,20,21]. In the present paper, an extension of the original SIR model is studied. It accounts for HIV prevalence, TB–HIV co-infection, and death components. We use the data obtained from 2012–2020 on TB and HIV cases to derive parameter values. The fitted model is then applied for a 30-year forward-in-time numerical simulation of the co-infection flow dynamics specific to the population of South Africa.

This paper is organized as follows. In Section 2, we formulate TB–HIV co-infection transmission dynamics using the SIRD model. In Section 3, the model parameter values for and results of the analysis are presented for a forward-in-time numerical simulation. The impact of the results obtained is elaborated in detail in Section 4. A summary of the research work, implications for policy and practice, limitations, and future research is given in Section 5. 

## 2. Materials and Methods

### 2.1. Model Specification

**Figure 1:**
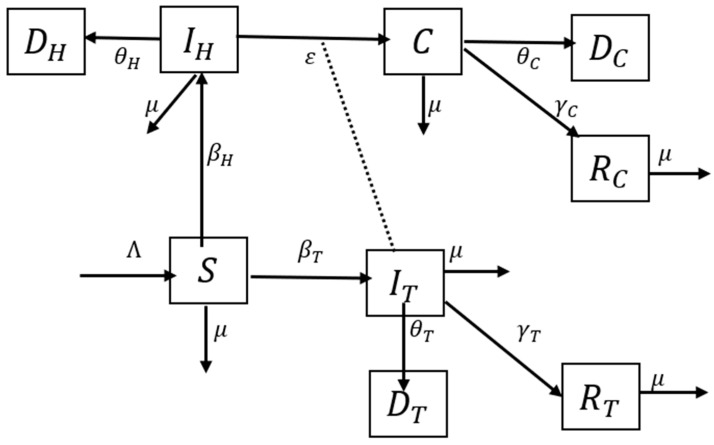
Deterministic SIRD for TB–HIV co-infection model. S represents susceptible individuals, $I_{T}$ represents TB-infected individuals, $I_{H}$ represents HIV-infected individuals, C represents TB–HIV-co-infected individuals, $R_{T}$ represents TB-recovered individuals, $R_{C}$ represents TB-recovered individuals with HIV infection, $D_{T}$ represents TB death, $D_{C}$ represents TB–HIV co-infection death, and $D_{H}$ represents HIV death.

The compartmental structure of this model utilizes the available data on TB (World Health Organization (WHO), StopTB Partnership) and HIV (Joint United Nations Programme on HIV and AIDS (UNAIDS)) in South Africa from 2012–2020, shown in Table 1, Table 2 and Table 3, to describe the TB–HIV co-infection flow dynamics. The Susceptible (S)–Infectious (I)–Recovered (R)–Dead (D) TB–HIV co-infection model is illustrated in Figure 1. The model subdivides the human population into nine (9) mutually exclusive compartments, namely, susceptible individuals (*S*), TB infected ($I_{T}$), HIV infected ($I_{H}$), TB–HIV co-infected (*C*), TB-recovered individuals ($R_{T}$), TB-recovered individuals with HIV infection ($R_{C}$), TB death ($D_{T}$), TB–HIV co-infection death ($D_{C}$), and HIV death ($D_{H}$).

The total population at time t, denoted by $N\left(t\right),$ is given by:

(1) $$N\left(t\right)={S\left(t\right)+I}_{T}\left(t\right)+I_{H}\left(t\right)+C\left(t\right)+R_{T}\left(t\right)+R_{C}\left(t\right)+D_{T}\left(t\right)+D_{C}\left(t\right)+D_{H}\left(t\right),$$

The susceptible population is increased at recruitment rate *λ* and reduced as a result of interaction with TB-infected individuals or HIV-infected individuals at the rate $\beta_{T}$ and $\beta_{H}$, respectively, which leads to an increase in both the TB and HIV classes. All individuals suffer from natural death at a constant rate μ. The HIV class is reduced by disease-induced death at the rate $\theta_{H}$. The TB class is reduced as a result of recovery at the rate $\gamma_{T}$, disease-induced death at the rate $\theta_{T}$, and interaction with HIV-infected individuals at the rate *ε*, which leads to an increase in the co-infection class. The co-infection class is reduced as a result of disease-induced death at the rate $\theta_{C}$ and recovery from TB infection at the rate $\gamma_{C}$. TB re-infection is possible and does occur; however, for simplicity of the model, it is assumed that recovered individuals are immune and no longer susceptible to re-infection. The aforementioned assumption results in the system of differential equations given in Equation (2) that describe the transmission dynamics of TB–HIV co-infection.

Using the year 2012 as a baseline for the model, we write the initial conditions as S(0)=N−383,000−63,000,000−677,000, $I_{T}\left(0\right)=383,000,\;I_{H}(0)=6,300,000,$ and C(0)=677,000. The argument goes as follows. It is assumed that in an infinitesimally small interval [t, t+dt], the probability of a susceptible individual being infected with TB is $\frac{I_{T}\left(t\right)}{N}$, while the probability of being infected with HIV is $\frac{I_{H}\left(t\right)}{N}$. It is assumed that a TB-infected individual passes on the disease with a probability $\beta_{T}$, while an HIV-infected individual passes on the virus with a probability $\beta_{H}$. Thus, the chance for a susceptible individual to become infected is $\beta_{T}\frac{I_{T}\left(t\right)}{N}$, and, on average, $\beta_{T}\frac{S\left(t\right)I_{T}\left(t\right)}{N}$ susceptible individuals become infected. The same goes for HIV infection, which happens with a probability $\beta_{H}\frac{I_{H}\left(t\right)}{N}$, and the mean number of susceptible individuals who catch an HIV infection is $\beta_{H}\frac{S\left(t\right)I_{H}\left(t\right)}{N}$. 

Additionally, it is assumed that each TB-infected individual will recover with probability $\gamma_{T}$ or die with probability $\theta_{T}$, and so, on average, $\gamma_{T}I_{T}\left(t\right)$ TB-infected individuals will leave the TB-infected compartment and move to the TB-recovered compartment (HIV negative). In addition, on average, $\theta_{T}I_{T}\left(t\right)$ TB-infected individuals will leave the TB-infected compartment due to death from the infection and move to the TB death compartment. 

Exit from the HIV compartment is only through death with a probability $\theta_{H}$, and an average of $\theta_{H}I_{H}\left(t\right)$, since there is currently no cure for the virus. This exit is from the HIV compartment to the HIV death compartment. Co-infection with TB and HIV occurs with probability *ε*, and so, on average, $\varepsilon I_{T}{\left(t\right)I}_{H}\left(t\right)$ individuals leave the HIV compartment and move to the co-infected compartment. 

A co-infected individual will recover from TB alone (since there is currently no cure for HIV) with a probability $\gamma_{C}$ or die with probability $\theta_{C},$ and so, on average, $\gamma_{C}C\left(t\right)$ TB–HIV-co-infected individuals will leave the co-infected compartment and move to the TB-recovered compartment (HIV positive). Additionally, on average, $\theta_{C}C\left(t\right)$ TB–HIV-co-infected individuals will leave the co-infected compartment due to death from the co-infection and move to the death compartment due to co-infection.

(2) $$\left\{\begin{array}{c} \frac{dS\left(t\right)}{dt}=\lambda N-\frac{\beta_{T}I_{T}\left(t\right)S\left(t\right)}{N}-\frac{\beta_{H}I_{H}\left(t\right)S\left(t\right)}{N}-\mu S\left(t\right),\;\;\;\;\;\;\;\;\;\;\;\;\;\;\;\;\;\;\;\;\;\;\;\;\;\\ \frac{dI_{T}\left(t\right)}{dt}=\frac{\beta_{T}I_{T}\left(t\right)S\left(t\right)}{N}-\left(\gamma_{T}+\theta_{T}+\mu \right)I_{T}\left(t\right),\\ \frac{dI_{H}\left(t\right)}{dt}=\frac{\beta_{H}I_{H}\left(t\right)S\left(t\right)}{N}-\varepsilon I_{T}{\left(t\right)I}_{H}\left(t\right)-\left(\theta_{H}+\mu \right)I_{H}\left(t\right),\;\;\;\;\;\;\;\;\;\;\;\;\;\;\;\;\;\;\;\;\;\;\;\;\;\;\;\;\;\;\;\;\;\;\;\;\;\;\;\;\;\;\;\;\;\\ \frac{dC\left(t\right)}{dt}=\varepsilon I_{T}{\left(t\right)I}_{H}\left(t\right)-\left(\gamma_{C}+\theta_{C}+\mu \right)C\left(t\right),\;\;\;\;\;\;\;\;\;\;\;\;\;\;\;\;\;\;\;\;\;\;\;\;\;\;\;\;\;\;\;\;\\ \frac{dR_{T}\left(t\right)}{dt}=\gamma_{T}I_{T}\left(t\right)-\mu R_{T}(t),\;\;\;\;\;\;\;\;\;\;\;\;\;\;\;\;\;\;\;\;\;\;\;\;\;\;\;\;\;\;\;\;\;\;\;\;\;\;\;\;\;\;\;\;\;\;\;\;\;\;\;\;\;\;\;\;\;\;\;\;\;\;\;\;\;\;\;\;\;\;\;\;\;\;\\ \frac{dR_{C}\left(t\right)}{dt}=\gamma_{C}C\left(t\right)-\mu R_{C}(t),\;\;\;\;\;\;\;\;\;\;\;\;\;\;\;\;\;\;\;\;\;\;\;\;\;\;\;\;\;\;\;\;\;\;\;\;\;\;\;\;\;\;\;\;\;\;\;\;\;\;\;\;\;\;\;\;\;\;\;\;\;\;\;\;\;\;\;\;\;\;\;\;\;\;\\ \frac{dD_{T}\left(t\right)}{dt}=\theta_{T}I_{T}\left(t\right),\;\;\;\;\;\;\;\;\;\;\;\;\;\;\;\;\;\;\;\;\;\;\;\;\;\;\;\;\;\;\;\;\;\;\;\;\;\;\;\;\;\;\;\;\;\;\;\;\;\;\;\;\;\;\;\;\;\;\;\;\;\;\;\;\;\;\;\;\;\;\;\;\;\;\\ \frac{dD_{H}\left(t\right)}{dt}=\theta_{H}I_{H}\left(t\right),\;\;\;\;\;\;\;\;\;\;\;\;\;\;\;\;\;\;\;\;\;\;\;\;\;\;\;\;\;\;\;\;\;\;\;\;\;\;\;\;\;\;\;\;\;\;\;\;\;\;\;\;\;\;\;\;\;\;\;\;\;\;\;\;\;\;\;\;\;\;\;\;\;\\ \frac{dD_{C}\left(t\right)}{dt}=\theta_{C}C\left(t\right).\;\;\;\;\;\;\;\;\;\;\;\;\;\;\;\;\;\;\;\;\;\;\;\;\;\;\;\;\;\;\;\;\;\;\;\;\;\;\;\;\;\;\;\;\;\;\;\;\;\;\;\;\;\;\;\;\;\;\;\;\;\;\;\;\;\;\;\;\;\;\;\; \end{array}\right.$$

### 2.2. Parameter Estimation

Consider discrete time points a unit interval apart (1, 2, 3, etc.). The above differential equations in Equation (2) turn into the difference equations for *j* = 0, 1, 2, ….

(3) $$\left\{\begin{array}{c} S\left(j+1\right)=S\left(j\right)+\lambda N-\frac{\beta_{T}I_{T}\left(j\right)S\left(j\right)}{N}-\frac{\beta_{H}I_{H}\left(j\right)S\left(j\right)}{N}-\mu S\left(j\right),\;\;\;\;\;\;\;\;\;\;\;\;\;\;\;\;\;\;\;\;\;\;\;\;\;\;\;\\ I_{T}\left(j+1\right)=I_{T}\left(j\right)+\frac{\beta_{T}I_{T}\left(j\right)S\left(j\right)}{N}-\left(\gamma_{T}+\theta_{T}+\mu \right)I_{T}\left(j\right),\\ I_{H}\left(j+1\right)=I_{H}\left(j\right)+\frac{\beta_{H}I_{H}\left(j\right)S\left(j\right)}{N}-\varepsilon I_{T}{\left(j\right)I}_{H}\left(j\right)-\left(\theta_{H}+\mu \right)I_{H}\left(j\right),\;\;\;\;\;\;\;\;\;\;\;\;\;\;\;\;\;\;\;\;\;\;\;\;\;\;\;\;\;\;\;\;\;\;\;\;\;\;\;\;\;\;\;\\ C\left(j+1\right)=C\left(j\right)+\varepsilon I_{T}{\left(j\right)I}_{H}\left(j\right)-\left(\gamma_{C}+\theta_{C}+\mu \right)C\left(j\right),\;\;\;\;\;\;\;\;\;\;\;\;\;\;\;\;\;\;\;\;\;\;\;\;\;\;\;\;\;\;\;\\ R_{T}\left(j+1\right)=R_{T}\left(j\right)+\gamma_{T}I_{T}\left(j\right)-\mu R_{T}(j),\;\;\;\;\;\;\;\;\;\;\;\;\;\;\;\;\;\;\;\;\;\;\;\;\;\;\;\;\;\;\;\;\;\;\;\;\;\;\;\;\;\;\;\;\;\;\;\;\;\;\;\;\;\;\;\;\;\;\;\;\;\;\;\;\;\;\;\;\;\;\\ R_{C}\left(j+1\right)=R_{C}\left(j\right)+\gamma_{C}C\left(j\right)-\mu R_{C}(j),\;\;\;\;\;\;\;\;\;\;\;\;\;\;\;\;\;\;\;\;\;\;\;\;\;\;\;\;\;\;\;\;\;\;\;\;\;\;\;\;\;\;\;\;\;\;\;\;\;\;\;\;\;\;\;\;\;\;\;\;\;\;\;\;\;\;\;\;\;\;\;\;\\ D_{T}\left(j+1\right)={D_{T}\left(j\right)+\theta }_{T}I_{T}\left(j\right),\;\;\;\;\;\;\;\;\;\;\;\;\;\;\;\;\;\;\;\;\;\;\;\;\;\;\;\;\;\;\;\;\;\;\;\;\;\;\;\;\;\;\;\;\;\;\;\;\;\;\;\;\;\;\;\;\;\;\;\;\;\;\;\;\;\;\;\;\;\;\\ D_{H}\left(j+1\right)={D_{H}\left(j\right)+\theta }_{H}I_{H}\left(j\right),\;\;\;\;\;\;\;\;\;\;\;\;\;\;\;\;\;\;\;\;\;\;\;\;\;\;\;\;\;\;\;\;\;\;\;\;\;\;\;\;\;\;\;\;\;\;\;\;\;\;\;\;\;\;\;\;\;\;\;\;\;\;\;\;\;\;\;\;\;\\ D_{C}\left(j+1\right)=D_{C}\left(j\right)+\theta_{C}C\left(j\right).\;\;\;\;\;\;\;\;\;\;\;\;\;\;\;\;\;\;\;\;\;\;\;\;\;\;\;\;\;\;\;\;\;\;\;\;\;\;\;\;\;\;\;\;\;\;\;\;\;\;\;\;\;\;\;\;\;\;\;\;\;\;\;\;\;\;\;\;\; \end{array}\right.$$

Denoting the increments by $∆S\left(j\right)=S\left(j+1\right)-S\left(j\right),\;∆I_{T}\left(j\right)=I_{T}\left(j+1\right)-I_{T}\left(j\right),\;∆I_{H}\left(j\right)=I_{H}\left(j+1\right)-I_{H}\left(j\right),\;∆C\left(j\right)=C\left(j+1\right)-C\left(j\right),\;∆R_{T}\left(j\right)=R_{T}\left(j+1\right)-R_{T}\left(j\right),\;∆R_{C}\left(j\right)=R_{C}\left(j+1\right)-R_{C}\left(j\right),\;∆D_{T}\left(j\right)=D_{T}\left(j+1\right)-D_{T}\left(j\right),\;∆D_{H}\left(j\right)=D_{H}\left(j+1\right)-D_{H}\left(j\right),and\;∆D_{C}\left(j\right)=D_{C}\left(j+1\right)-D_{C}\left(j\right)$, the equations in Equation (3) can be rewritten as:

(4) $$\left\{\begin{array}{c} ∆S\left(j\right)+∆I_{T}\left(j\right)+∆R_{T}\left(j\right)+∆D_{T}\left(j\right)+∆I_{H}\left(j\right)+∆C\left(j\right)+∆R_{C}\left(j\right)+∆D_{C}\left(j\right)+∆D_{H}\left(j\right)+\\ \left(R_{T}\left(j\right)+I_{T}\left(j\right)+R_{C}\left(j\right)+C\left(j\right)+I_{H}\left(j\right)+S(j)\right)\mu =\lambda N,\\ \frac{∆I_{T}\left(j\right)+∆R_{T}\left(j\right)+∆D_{T}\left(j\right)+\left(R_{T}\left(j\right)+I_{T}\left(j\right)\right)\mu }{S\left(j\right)I_{T}\left(j\right)}=\frac{\beta_{T}}{N},\\ \frac{∆I_{H}\left(j\right)+∆C\left(j\right)+∆R_{C}\left(j\right)+∆D_{C}\left(j\right)+∆D_{H}\left(j\right)+\left(R_{C}\left(j\right)+C\left(j\right)+I_{H}\left(j\right)\right)\mu }{S\left(j\right)I_{H}\left(j\right)}=\frac{\beta_{H}}{N},\\ \frac{∆C\left(j\right)+∆R_{C}\left(j\right)+∆D_{C}\left(j\right)+\left(R_{C}\left(j\right)+C(j)\right)\mu }{I_{T}{\left(j\right)I}_{H}\left(j\right)}=\varepsilon ,\\ \frac{\;∆R_{T}\left(j\right)+\mu R_{T}\left(j\right)}{I_{T}\left(j\right)}=\gamma_{T},\\ \frac{\;∆R_{C}\left(j\right)+\mu R_{C}\left(j\right)}{C\left(j\right)}=\gamma_{C},\\ \frac{\;∆D_{T}\left(j\right)}{I_{T}\left(j\right)}=\theta_{T},\\ \frac{\;∆D_{H}\left(j\right)}{I_{H}\left(j\right)}=\theta_{H},\\ \frac{\;∆D_{C}\left(j\right)}{C(j)}=\theta_{C}. \end{array}\right.$$

From these equations in Equation (4), it follows that the parameters *λ*, $\beta_{T}$, $\beta_{H}$, *ε*, $\gamma_{T}$, $\gamma_{C}$, $\theta_{T}$, $\theta_{H}$, and $\theta_{C}$ can be estimated by the method of moments, according to which:

(5) $${\hat{\lambda }}_{MM}=\frac{1}{N}\left(sample\;mean\;of\left(\begin{array}{c} ∆S\left(j\right)+∆I_{T}\left(j\right)+∆R_{T}\left(j\right)+∆D_{T}\left(j\right)+∆I_{H}\left(j\right)\\ +∆C\left(j\right)+∆R_{C}\left(j\right)+∆D_{C}\left(j\right)+∆D_{H}\left(j\right)\\ +\left(R_{T}\left(j\right)+I_{T}\left(j\right)+R_{C}\left(j\right)+C\left(j\right)+I_{H}(j)+S(j)\right)\mu \end{array}\right)\right),\;{{\hat{\beta }}_{T}}_{MM}=N\left(sample\;mean\;of\left(\frac{∆I_{T}\left(j\right)+∆R_{T}\left(j\right)+∆D_{T}\left(j\right)+\left(R_{T}\left(j\right)+I_{T}\left(j\right)\right)\mu }{S\left(j\right)I_{T}\left(j\right)}\right)\right),\;{{\hat{\beta }}_{H}}_{MM}=N\left(sample\;mean\;of\left(\frac{\begin{array}{c} \;∆I_{H}\left(j\right)+∆C\left(j\right)+∆R_{C}\left(j\right)+∆D_{C}\left(j\right)+∆D_{H}\left(j\right)\\ +\left(R_{C}\left(j\right)+C\left(j\right)+I_{H}\left(j\right)\right)\mu \end{array}}{S\left(j\right)I_{H}\left(j\right)}\right)\right),\;{\hat{\varepsilon }}_{MM}=sample\;mean\;of\left(\frac{∆C\left(j\right)+∆R_{C}\left(j\right)+∆D_{C}\left(j\right)+\left(R_{C}\left(j\right)+C(j)\right)\mu }{I_{T}{\left(j\right)I}_{H}\left(j\right)}\right),\;{{\hat{\gamma }}_{T}}_{MM}=sample\;mean\;of\left(\frac{\;∆R_{T}\left(j\right)+\mu R_{T}(j)}{I_{T}\left(j\right)}\right),\;\;{{\hat{\gamma }}_{C}}_{MM}=sample\;mean\;of\left(\frac{\;∆R_{C}\left(j\right)+\mu R_{C}(j)}{C\left(j\right)}\right),\;{{\hat{\theta }}_{T}}_{MM}=sample\;mean\;of\left(\frac{\;∆D_{T}\left(j\right)}{I_{T}\left(j\right)}\right),\;{{\hat{\theta }}_{H}}_{MM}=sample\;mean\;of\left(\frac{\;∆D_{H}\left(j\right)}{I_{H}\left(j\right)}\right),\;\;and\;\;\;{{\hat{\theta }}_{C}}_{MM}=sample\;mean\;of\left(\frac{\;∆D_{C}\left(j\right)}{C\left(j\right)}\right).$$

### 2.3. Disease-Free Equilibrium

The disease-free equilibrium is the point where there is no disease present in the population. In mathematical terms, the equilibrium point is a constant solution to the system of differential equations which is found by setting the derivatives on the left-hand side of Equation (2) equal to zero. Here, we consider two sub-models that have well-defined disease-free equilibrium points: the TB-only and HIV-only models. 

For the TB-only model, consider the sub-model of Equation (2) with no HIV infection given by:

(6) $$\left\{\begin{array}{c} \frac{dS\left(t\right)}{dt}=\lambda N-\frac{\beta_{T}I_{T}\left(t\right)S\left(t\right)}{N}-\mu S\left(t\right),\\ \frac{dI_{T}\left(t\right)}{dt}=\frac{\beta_{T}I_{T}\left(t\right)S\left(t\right)}{N}-\left(\gamma_{T}+\theta_{T}+\mu \right)I_{T}\left(t\right),\\ \frac{dR_{T}\left(t\right)}{dt}=\gamma_{T}I_{T}\left(t\right)-\mu R_{T}(t),\\ \frac{dD_{T}\left(t\right)}{dt}=\theta_{T}I_{T}\left(t\right). \end{array}\right.$$

(7) $$N\left(t\right)={S\left(t\right)+I}_{T}\left(t\right)+R_{T}\left(t\right)+D_{T}\left(t\right).$$

Let $s=\frac{S}{N},i_{T}=\frac{I_{T}}{N},r_{T}=\frac{R_{T}}{N},and\;d_{T}=\frac{D_{T}}{N}$ represent the fractions of the susceptible, TB infected, TB recovered, and TB deaths in the population, respectively. Then, from Equation (7) we obtain:

(8) $$s(t){+i}_{T}\left(t\right)+r_{T}\left(t\right)+d_{T}\left(t\right)=1.$$

Thus, Equation (6) is reduced to:

(9) $$\left\{\begin{array}{c} \frac{d}{dt}s(t)=\lambda -\beta_{T}i_{T}\left(t\right)s\left(t\right)-\mu s\left(t\right),\\ \frac{d}{dt}i_{T}(t)=\beta_{T}i_{T}\left(t\right)s\left(t\right)-\left(\gamma_{T}+\theta_{T}+\mu \right)i_{T}\left(t\right),\\ \frac{d}{dt}r_{T}(t)=\gamma_{T}i_{T}\left(t\right)-\mu r_{T}(t),\\ \frac{d}{dt}{\;d}_{T}(t)=\theta_{T}i_{T}\left(t\right). \end{array}\right.$$

The disease-free equilibrium for the TB-only model is obtained as the solution of the equations in (9) with the left-hand sides replaced by zeros. The result is s*,iT*,rT*,dT*=(λμ,0, 0, 0).

For the HIV-only model, the sub-model of Equation (2) with no TB disease is given by:

(10) $$\left\{\begin{array}{c} \frac{dS\left(t\right)}{dt}=\lambda N-\frac{\beta_{H}I_{H}\left(t\right)S\left(t\right)}{N}-\mu S\left(t\right),\\ \frac{dI_{H}\left(t\right)}{dt}=\frac{\beta_{H}I_{H}\left(t\right)S\left(t\right)}{N}-\left(\theta_{H}+\mu \right)I_{H}\left(t\right),\\ \frac{dD_{H}\left(t\right)}{dt}=\theta_{H}I_{H}\left(t\right). \end{array}\right.$$

(11) $$N\left(t\right)={S\left(t\right)+I}_{H}\left(t\right)+D_{H}\left(t\right).$$

Let $s=\frac{S}{N},i_{H}=\frac{I_{H}}{N},and\;d_{H}=\frac{D_{H}}{N}$ represent the fractions of the susceptible, HIV infected, and HIV deaths in the population, respectively. Then, from Equation (11), we obtain:

(12) $$s(t){+i}_{H}\left(t\right)+d_{H}\left(t\right)=1.$$

Hence, Equation (10) is reduced to:

(13) $$\left\{\begin{array}{c} \frac{d}{dt}s\left(t\right)=\lambda -\beta_{T}i_{H}\left(t\right)s\left(t\right)-\mu s\left(t\right),\\ \frac{d}{dt}i_{H}(t)=\beta_{T}i_{H}\left(t\right)s\left(t\right)-\left(\theta_{H}+\mu \right)i_{H}\left(t\right)\\ \frac{d}{dt}d_{H}(t)=\theta_{H}i_{H}\left(t\right). \end{array}\right.$$

The disease-free equilibrium is obtained by setting to zero the left-hand sides of the equations in (13) and solving them. It is not difficult to see that the equilibrium point is s*,iH*,dH*=(λμ,0, 0).

### 2.4. Basic Reproduction Number

The basic reproduction number $R_{0}$ is a crucial metric that dictates the course that an epidemic will take in the future. It is defined as the average number of secondary infections that occur after an infected person is introduced into a population in which every person is susceptible to the disease. If $R_{0}>1,$ the infection can start spreading, but if $R_{0}<1,$ it is bound to die out.

The next-generation matrix approach by Driessche and Watmough [22] is applied to evaluate the basic reproduction number.

For the TB-only model, the nonlinear terms with the new infection F and the outflow term V are given by $F=\beta_{T}i_{T}\left(t\right)s\left(t\right)\;and\;V=\left(\gamma_{T}+\theta_{T}+\mu \right)i_{T}\left(t\right)$. The partial derivatives of F and V with respect to $i_{T}$ at the disease-free equilibrium s*,iT*,rT*,dT*=(λμ,0, 0, 0) are $F=\beta_{T}s^{*}=\frac{\lambda \beta_{T}}{\mu }$ and $V=\gamma_{T}+\theta_{T}+\mu$. The basic reproduction number is computed as:

(14) $$R_{0T}=FV^{-1}=\frac{\lambda \beta_{T}}{\mu \left(\gamma_{T}+\theta_{T}+\mu \right)}.$$

For the HIV-only model, the nonlinear terms with the new infection F and the outflow term V are given by $F=\beta_{T}i_{H}\left(t\right)s\left(t\right)\;and\;V=\left(\theta_{H}+\mu \right)i_{H}\left(t\right)$. The partial derivatives of F and V with respect to $i_{H}$ at the disease-free equilibrium s*,iH*,dH*=(λμ, 0, 0) are $F=\beta_{T}s^{*}=\frac{\lambda \beta_{H}}{\mu }\;and\;V=\theta_{H}+\mu$. The basic reproduction number is obtained as:

(15) $$R_{0H}=FV^{-1}=\frac{{\lambda \beta }_{H}}{\mu \left(\theta_{H}+\mu \right)}.$$

Finally, combining the results for the two sub-models, we see that the basic reproduction number $R_{0}$ of the full model in Equation (2) is given by [23]:

(16) $$R_{0}=\max\left\{R_{0T},R_{0H}\right\}=\max\left\{\frac{\lambda \beta_{T}}{\mu \left(\gamma_{T}+\theta_{T}+\mu \right)},\frac{{\lambda \beta }_{H}}{\mu \left(\theta_{H}+\mu \right)}\right\}.$$

## 3. Results

### 3.1. Parameter Estimates

The model parameters are estimated according to the expressions given in Equation (5). The estimated values are shown below in Table 4. The dataset and code used in the numerical computation can be found in the Supplementary File S1. For the estimate of the natural death rate μ, we use the value of 1/70=0.0143 given in [24]. 

Plugging the values listed in Table 4 into Equation (16), we estimate the basic reproduction number as ${\hat{R}}_{0}=\max\left\{1.69,5.47\right\}=5.47.$

### 3.2. Numerical Simulation

Numerical simulations were carried out using the R software to show the trajectories of the population in HIV-infected, HIV death, TB-infected, TB recovery, and TB death classes, subject to the given initial values and estimated parameters: $S\left(0\right)=45,785,033,\;I_{T}\left(0\right)=383,000,\;{\;I}_{H}\left(0\right)=6,300,000,\;C\left(0\right)=677,000,\;{\;R}_{T}\left(0\right)=0,\;{\;R}_{C}\left(0\right)=0,\;{\;D}_{T}\left(0\right)=0,\;{\;D}_{C}\left(0\right)=0,\;{\;D}_{H}\left(0\right)=0,\;\hat{\lambda }=0.0280,\;{\hat{\beta }}_{T}=0.3859,\;{\hat{\beta }}_{H}=0.0828,\;\hat{\varepsilon }=8.383e-08,{\hat{\gamma }}_{T}=0.3568,\;{\hat{\gamma }}_{C}=0.2654,\;{\hat{\theta }}_{T}=0.0767,\;{\hat{\theta }}_{H}=0.0154,{\hat{\theta }}_{C}=0.2096,$ and $\hat{\mu }=0.0143$.

**Figure 2:**
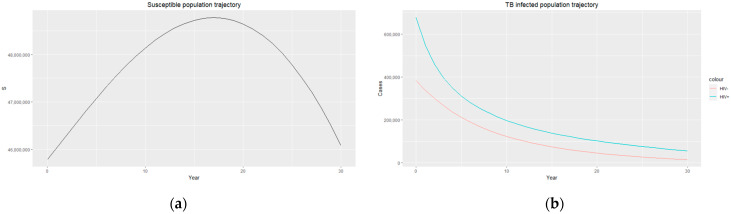
(**a**) Susceptible population trajectory; (**b**) TB-infected population trajectories for HIV-positive individuals (blue) and HIV-negative individuals (red).

**Figure 3:**
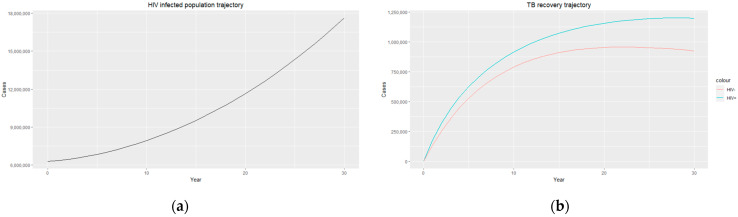
(**a**) HIV-infected population trajectory; (**b**) TB recovery trajectory for HIV-positive individuals (blue) and HIV-negative individuals (red).

**Figure 4:**
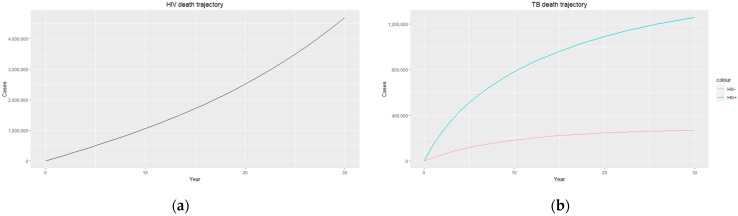
(**a**) HIV death trajectory; (**b**) TB death trajectories for HIV-positive individuals (blue) and HIV-negative individuals (red).

**Figure 5:**
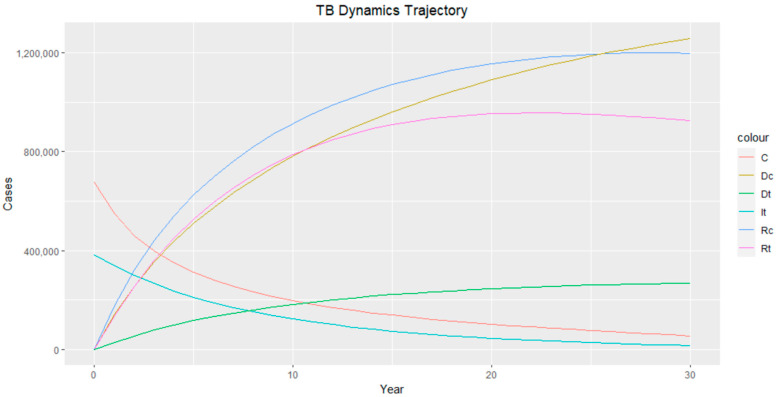
TB dynamics trajectory. Class C represents TB–HIV-co-infected individuals, $D_{C}$ represents TB–HIV co-infection death, $D_{T}$ represents TB death, $I_{T}$ represents TB-infected individuals, $R_{C}$ represents TB-recovered individuals with HIV infection, and $R_{T}$ represents TB-recovered individuals.

**Figure 6:**
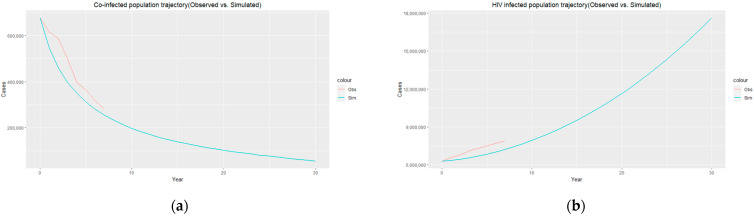
(**a**) Co-infected population trajectory (observed (red)) vs. simulated (blue)); (**b**) HIV-infected population trajectory (observed (red) vs. simulated (blue)).

The trajectories of the compartmental dynamics are shown in Figure 2, Figure 3 and Figure 4 below. The dynamics trajectory excluding the HIV compartments is shown in Figure 5. Figure 6 displays the observed vs. simulated data for the TB–HIV co-infection and HIV infection compartments. 

## 4. Discussion

The method of moments is utilized to estimate the values of the model parameters for data obtained from the WHO, UNAIDS, and StopTB Partnership. The data are given in Table 1, Table 2 and Table 3, and the results are presented in Table 4. The estimated basic reproduction numbers for TB-only and HIV-only models are 1.69 and 5.47, respectively. This shows that both infections are endemic in the population since $R_{0}>1$, with a person infected with HIV being more susceptible to contracting TB. Ensuring effective treatment for people living with HIV can help improve immune function, reducing the risk of developing TB. In addition, conducting contact tracing as a means to identify individuals who have been in close contact with TB patients, such as household contacts and healthcare workers, and providing them with the appropriate care can help reduce the likelihood of transmission.

The estimated parameters are used to perform a 30-year numerical simulation of the dynamics of the model using 2012 as the baseline, and the results are presented in Figure 2, Figure 3 and Figure 4. The numerical simulations agree with the data obtained from 2012–2020 and the likelihood of future trends (see Figure 6). Figure 2a shows a steady increase in the susceptible class for a while, which can be attributed to an increase in the population through migration and birth. However, over time, a steady decrease in the susceptible class is observed due to more people being infected with HIV. Figure 2b demonstrates that the numbers of HIV-negative and HIV-positive individuals both decrease in TB infection at similar rates due to effective treatment but do not die out over time consequent to the basic reproduction number being $R_{0}>1$. Essentially, infected individuals will still be present in the population at any given time irrespective of the effectiveness of treatment. From Figure 3b, it can be seen that HIV-positive individuals have a slightly higher recovery rate than HIV-negative individuals. We would expect the opposite given that people living with HIV have a weaker immune system; however, in South Africa, more than 60% of TB cases are co-infected with HIV, which could explain why greater recovery from TB is seen amongst people living with HIV. It is important to note that while HIV-positive individuals may have a slightly higher TB recovery rate, they are still at a higher risk of developing active TB and experiencing more severe TB symptoms compared to HIV-negative individuals. Therefore, it is crucial to prioritize early detection and timely treatment for both HIV-positive and HIV-negative individuals to control the spread of TB and improve overall health outcomes.

Figure 4b shows a sharp contrast in TB death for both HIV-positive and HIV-negative individuals, with people living with HIV having a much higher TB death rate, which could be due to HIV-positive individuals being co-infected with other life-threatening diseases and, in most cases, the development of Acquired Immunodeficiency Syndrome (AIDS). The weakened immune system caused by HIV/AIDS makes individuals more vulnerable to severe forms of TB and complicates their treatment outcomes, resulting in higher mortality rates. Prioritising a comprehensive and integrated approach to TB and HIV care, including access to antiretroviral therapy (ART), preventive measures, and coordinated healthcare services, will help to improve survival rates and enhance the overall well-being of individuals living with HIV and TB co-infection.

Figure 3a and Figure 4a display an exponential increase in both HIV-infected individuals and HIV deaths over time, with the prevalence of HIV infection reaching almost 18 million, indicating a significant burden of the disease within the population. Similarly, the number of HIV recorded deaths reaches close to 5 million, highlighting the impact of HIV on mortality rates in the country. This increase in HIV infection and death rates highlights the urgent need for comprehensive prevention, testing, and treatment strategies to address the HIV epidemic in South Africa. Efforts should focus on implementing robust prevention programs that prioritize education and awareness about HIV transmission, safe sex practices, and access to preventive measures such as condoms and pre-exposure prophylaxis (PrEP). Empowering individuals with adequate knowledge and resources can reduce the incidence of new HIV infections and promote healthier behaviours. Additionally, early diagnosis and timely initiation of ART for people living with HIV are essential as they improve immune function and reduce the risk of HIV-related deaths. It is essential to continue investing in research, innovation, and evidence-based interventions to effectively combat the HIV epidemic and reduce the number of HIV-related deaths in the country.

## 5. Conclusions

The co-infection of TB and HIV is a common health burden in South Africa. People living with HIV are significantly more likely to develop active TB disease than those without HIV. Conversely, TB can accelerate the progression of HIV to AIDS. To describe the flow dynamics of TB–HIV co-infection specific to the population of South Africa, we formulated a mathematical model using available TB and HIV data from 2012–2020. Parameter values in the model were estimated using the method of moments to make projections for a 30-year period using 2012 as the baseline. The basic reproduction number of the model was obtained, which showed that both TB and HIV are endemic in the population ($R_{0}>1$), with a person infected with HIV being more at risk of contracting TB.

Most people infected with TB can fully recover with the appropriate care, and South Africa has made significant progress in fighting the disease. This is also supported by our analysis, which showed a reduction in TB infection over time. However, challenges remain, particularly with the co-epidemic of TB and HIV. Findings from our analysis revealed a sharp contrast in TB-induced death in the population, with people living with HIV having a much higher TB death rate than those without HIV. Both diseases can progress more rapidly when coexisting, leading to higher morbidity and mortality rates. Furthermore, our projections indicated an exponential increase in HIV infection and death leaning towards 18 million people living with HIV and about 5 million recorded HIV-induced deaths.

To address the dual burden of TB/HIV and achieve better outcomes for infected individuals, TB policies and practices must be integrated with HIV care. HIV can carry a stigma, which may discourage people from seeking treatment or disclosing their status. Community education, awareness campaigns, and legal protections against discrimination can help establish a supportive environment for people living with HIV and TB. Enhancing the understanding of HIV transmission dynamics, treatment strategies, and prevention interventions through research and innovation needs to be prioritized. This includes supporting studies on new prevention technologies, vaccine development, and implementation science research. In addition, engaging communities, including people living with HIV and TB, in the design and implementation of interventions can play a significant role in providing support and advocacy for affected individuals and their families, as well as help to address barriers to care and ensure the sustainability of interventions.

Having obtained some useful insights from the modeling of transmission dynamics of TB–HIV co-infection in South Africa, it is necessary to note its limitations. The model relied on certain assumptions which did not fully capture the complex and varied realities of co-infection dynamics. Due to lack of data availability, assumptions were made to exclude TB re-infection in the population, as well as ART for people living with HIV. Factors such as healthcare infrastructure, social determinants, and demographic characteristics can also significantly influence transmission dynamics. Future research can investigate the impact of ART on TB transmission dynamics and outcomes. This includes studying the effect of ART on reducing the risk of developing active TB, the impact on TB treatment outcomes, and the potential for HIV treatment to indirectly reduce TB transmission by improving immune function. The impact of comorbidities, such as non-communicable diseases (NCDs), on the health outcomes of people living with HIV is also worth exploring. Understanding the interactions between HIV, TB, and NCDs can inform integrated approaches to care that address the multiple health needs of affected individuals.

By adopting a multi-faceted and integrated approach that combines prevention, testing, treatment, stigma reduction, research, and community engagement, South Africa can make significant progress in reducing HIV infection rates, decreasing HIV-related deaths, and mitigating the impact of TB–HIV co-infection. These efforts will contribute to better public health outcomes and improve the quality of life for individuals affected by these diseases.

## Figures and Tables

**Table 1:** The number of people living in South Africa with TB alone, HIV, and TB–HIV co-infection by year.

Year	TB	TB–HIV Co-Infection	HIV
2012	383,000	677,000	6,300,000
2013	383,000	615,000	6,600,000
2014	377,000	588,000	6,800,000
2015	382,000	500,000	7,100,000
2016	273,000	398,000	7,300,000
2017	237,000	363,000	7,500,000
2018	223,000	319,000	7,700,000
2019	203,000	282,000	7,900,000
2020	132,000	313,000	8,000,000

Data from WHO and UNAIDS. https://www.who.int/teams/global-tuberculosis-programme/data accessed on 24 March 2023; https://www.unaids.org/en/regionscountries/countries/southafrica accessed on 24 March 2023.

**Table 2:** The number of people in South Africa who died due to TB alone, HIV, and TB–HIV co-infection by year.

Year	TB Death	TB–HIV Co-Infection Death	HIV Death
2012	23,000	131,000	160,000
2013	22,000	117,000	130,000
2014	21,000	111,000	120,000
2015	21,000	100,000	110,000
2016	22,000	90,000	100,000
2017	22,000	78,000	94,000
2018	22,000	72,000	81,000
2019	23,000	67,000	72,000
2020	23,000	66,000	67,000

Data from WHO and UNAIDS. https://www.who.int/teams/global-tuberculosis-programme/data; https://www.unaids.org/en/regionscountries/countries/southafrica accessed on 24 March 2023.

**Table 3:** The number of people in South Africa who recovered from TB alone and TB–HIV co-infection by year.

Year	TB Recovery	TB–HIV Recovery
2012	105,000	148,000
2013	107,000	145,000
2014	108,000	140,000
2015	104,000	133,000
2016	87,000	107,000
2017	84,000	101,000
2018	99,000	63,000
2019	94,000	77,000
2020	87,000	90,000

Data from StopTB Partnership. https://www.stoptb.org/static_pages/ZAF_Dashboard.html accessed on 24 March 2023.

**Table 4:** Parameter notation and values of estimates.

Parameter	Estimated Value
Recruitment rate (*λ*)	$0.0280\;{person}^{-1}\;{year}^{-1}$
$\mathrm{TB}\;\mathrm{infection}\;\mathrm{rate}\;(\beta_{T}$)	$0.3859\;{person}^{-1}\;{year}^{-1}$
$\mathrm{HIV}\;\mathrm{infection}\;\mathrm{rate}\;(\beta_{H}$)	$0.0828\;{person}^{-1}\;{year}^{-1}$
TB–HIV co-infection rate (*ε*)	${8\times 10}^{-8}$ ${person}^{-1}\;{year}^{-1}$
$\mathrm{HIV}-\mathrm{negative}\;\mathrm{TB}\;\mathrm{recovery}\;\mathrm{rate}\;(\gamma_{T}$)	$0.3568\;{year}^{-1}$
$\mathrm{HIV}-\mathrm{positive}\;\mathrm{TB}\;\mathrm{recovery}\;\mathrm{rate}\;(\gamma_{C}$)	$0.2654\;{year}^{-1}$
$\mathrm{TB}\;\mathrm{infection}\;\mathrm{death}\;\mathrm{rate}\;(\theta_{T}$)	$0.0767\;{year}^{-1}$
$\mathrm{HIV}\;\mathrm{death}\;\mathrm{rate}\;(\theta_{H}$)	$0.0154\;{year}^{-1}$
$\mathrm{TB}–\mathrm{HIV}\;\mathrm{death}\;\mathrm{rate}\;(\theta_{C}$) Natural death rate (μ)	$0.2096\;{year}^{-1}$ $0.0143\;{year}^{-1}$

## Data Availability

The data presented in this study are available in Supplementary File S1.

## Supplementary Materials

The following supporting information can be downloaded at: https://www.mdpi.com/article/10.3390/epidemiologia4040036/s1, Supplementary File S1: Simdata, TB-HIV code.
